# Impact of Inosine on Chronic Unpredictable Mild Stress-Induced Depressive and Anxiety-Like Behaviors With the Alteration of Gut Microbiota

**DOI:** 10.3389/fcimb.2021.697640

**Published:** 2021-09-14

**Authors:** Xueer Liu, Teng Teng, Xuemei Li, Li Fan, Yajie Xiang, Yuanliang Jiang, Kang Du, Yuqing Zhang, Xinyu Zhou, Peng Xie

**Affiliations:** ^1^Department of Neurology, The First Affiliated Hospital of Chongqing Medical University, Chongqing, China; ^2^National Health Commission Key Laboratory of Diagnosis and Treatment on Brain Functional Diseases, The First Affiliated Hospital of Chongqing Medical University, Chongqing, China; ^3^Chongqing Key Laboratory of Neurobiology, Chongqing, China; ^4^Department of Psychiatry, The First Affiliated Hospital of Chongqing Medical University, Chongqing, China; ^5^Department of Neurology, The Second Affiliated Hospital of Chongqing Medical University, Chongqing, China

**Keywords:** depression, inosine, gut microbiota, chronic unpredictable mild stress, adolescent mice

## Abstract

Current antidepressants do not confer a clear advantage in children and adolescents with major depressive disorder (MDD). Accumulating evidence highlights the potential antidepressant-like effects of inosine on adult MDD, and gut microbiomes are significantly associated with MDD *via* the microbiota-gut-brain axis. However, few studies have investigated possible associations between inosine and gut microbiota in adolescents with MDD. The current study investigated the potential antidepressant effects of inosine in adolescent male C57BL/6 mice. After 4 weeks of chronic unpredictable mild stress (CUMS) stimulation, the mice were assessed by body weight, the sucrose preference test (SPT), open field test, and the elevated plus maze (EPM). The microbiota compositions of feces were determined by 16S rRNA gene sequencing. Inosine significantly improved CUMS-induced depressive and anxiety-like behaviors in adolescent mice including SPT and EPM results. Fecal microbial composition differed in the CON+saline, CUMS+saline, and CUMS+inosine groups, which were characterized by 126 discriminative amplicon sequence variants belonging to *Bacteroidetes* and *Firmicute* at the phylum level and *Muribaculaceae* and *Lachnospiraceae* at the family level. *Muribaculaceae* was positively associated with depressive and anxiety-like behaviors. KEGG functional analysis suggested that inosine might affect gut microbiota through carbohydrate metabolism and lipid metabolism pathways. The results of the study indicated that inosine improved depressive and anxiety-like behaviors in adolescent mice, in conjunction with the alteration of fecal microbial composition. Our findings may provide a novel perspective on the antidepressant effects of inosine in children and adolescents.

## Introduction

Major depressive disorder (MDD) is becoming a common mental health problem in young people, especially those aged 10–24 years (Mokdad et al., 2016). The prevalence of MDD in adolescents (aged 13–18 years) is approximately 11% in the USA (Avenevoli et al., 2015). Adolescents with MDD are prone to serious physical and mental health dysfunctions including high rates of substance abuse, smoking, obesity, and suicidal tendency (Hasler et al., 2005; Keenan-Miller et al., 2007). However, the majority of available antidepressants that are effective in adult patients with MDD have no significant effects in children and adolescents with MDD (Cipriani et al., 2018) and may also increase the risk of suicide in those young population (Cipriani et al., 2016). Thus, new effective and safe antidepressants need to be developed for use in young people with MDD.

In recent years, numerous studies have provided evidence of significant associations between inosine and MDD. Dysregulated purine metabolism with lower level of inosine was detected in MDD patients compared with healthy adults (Ali-Sisto et al., 2016). Similar results were obtained in our previous metabolic profiling research, in which the plasma level of inosine in children and adolescents with MDD was significantly lower than in healthy controls but recovered after treating with antidepressants (Zhou et al., 2019). Inosine is an endogenous purine nucleoside generated by adenosine deaminase (Junqueira et al., 2017) that showed antidepressant effects in adult depressive rodent models (Kaster et al., 2013; Muto et al., 2014). It also reduced immobility time in normal adolescent animals without stress stimuli, which activated extracellular signal-regulated kinases and cyclic AMP response element binding protein signaling pathways (Yuan et al., 2018). Inosine reportedly plays a role in depression through other signaling pathways, including the activation of mammalian target of rapamycin (Goncalves et al., 2017b), phosphoinositide 3-kinase signaling pathways, and the inhibition of glycogen synthase kinase 3beta (Goncalves et al., 2017a). Collective studies have thus demonstrated that inosine has marked antidepressant-like effects in adult animals, but no corresponding evidence has been generated in depressive adolescent animals.

Emerging evidence suggests that the underlying pathogenesis of MDD in depressive animals and humans involves dysbiosis of gut microbiota, and the effect of the gut-brain axis (Jiang et al., 2015; Zheng et al., 2016; Yu et al., 2017). Fluoxetine (Lukic et al., 2019; Sun et al., 2019), ketamine (Qu et al., 2017), escitalopram, and duloxetine (Lukic et al., 2019) can improve depressive and anxiety-like behaviors and are associated with alterations in gut microbiota composition. It was recently reported that the downregulation of inosine in the hippocampus of rats caused by depression was corrected after treatment with traditional Chinese medicine, and that these variations in inosine were eliminated after antibiotic intervention (Yu et al., 2020). The serum metabolite level of inosine was restored by *Lactobacillus* treatment in mice with gut microbial dysbiosis (He et al., 2017). The above results suggest that there may be an association between specific bacteria and inosine in MDD. The current study investigated the potential antidepressant effects of inosine in adolescent male mice with chronic unpredictable mild stress (CUMS), and related associations with gut microbiota.

## Material and Methods

### Animals and Ethics Statement

All male C57BL/6 mice (9–14 g) aged postnatal day (PND) 21 were purchased from the animal facility at Chongqing Medical University (Chongqing, China) and individually housed in standard cages under controlled temperature (22°C ± 1°C), relative humidity (50% ± 5%), and a 12-h light/dark cycle (lights on from 19:00 to 07:00). Food and water were provided *ad libitum*, except in the food and water deprivation stress condition. All animal procedures were approved by the Animal Ethics Committee of Chongqing Medical University, Chongqing, China (approval number 2017013), and all efforts were made to minimize animal suffering throughout the experiments.

### Experiment Schedule

**Figure 1:**
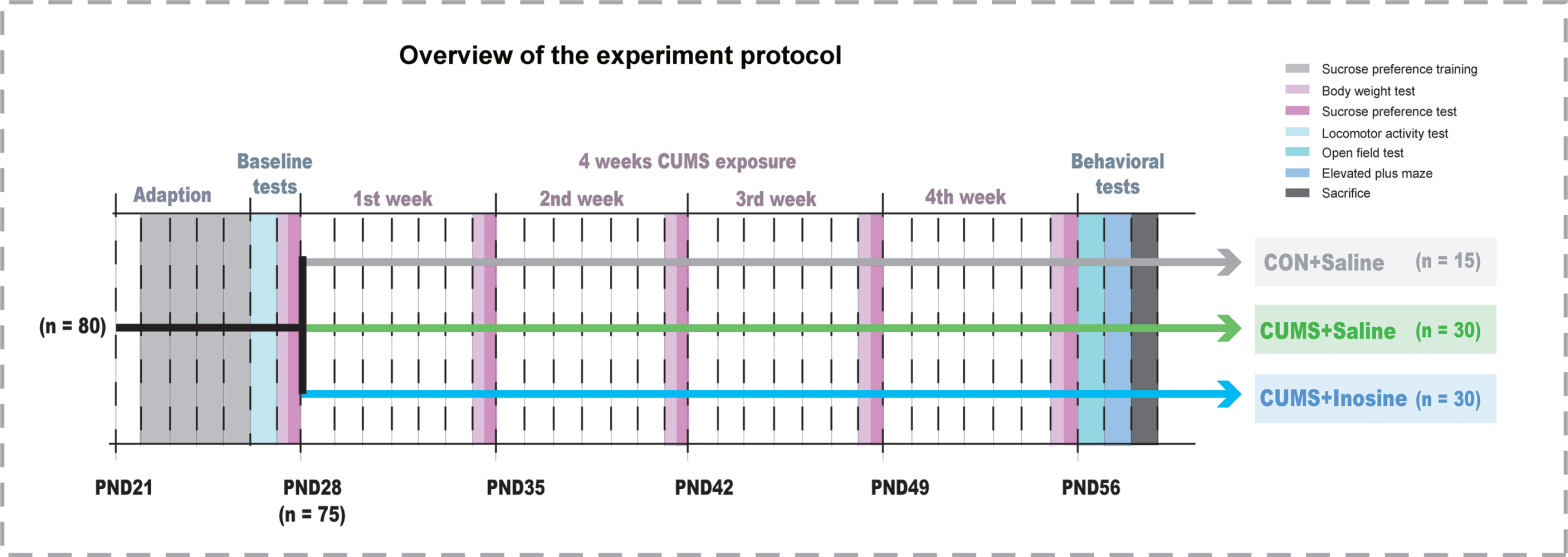
Experimental design of this study.

Eighty mice aged PND 21 were acclimatized for 1 week to standard experimental conditions before the commencement of the CUMS stimulation. Mice exhibiting baseline values below the 95% reference interval in the locomotor activity test (LAT) and the sucrose preference test (SPT) were regarded as outliers to be excluded. The remaining 75 mice were randomly assigned to three groups: CUMS+inosine group (*n* = 30, CUMS+intraperitoneally administered inosine), a CUMS+saline group (*n* = 30, CUMS+intraperitoneally administered saline), and a saline control group (CON+saline; *n* = 15, intraperitoneally administered saline). Body weight and SPT were measured at the end of each week, and the open field test (OFT) and elevated plus maze (EPM) were performed at the end of the study period. Inosine (Sigma-Aldrich, St Louis, Missouri, USA) was dissolved in 1.0% DMSO and 0.9% NaCl (1 mg/ml), and freshly prepared before intraperitoneal administration to the CUMS+inosine group at a dose of 10 mg/kg of body weight. CON+saline and CUMS+saline groups received corresponding intraperitoneal doses of sterile saline (1.0% DMSO and 0.9% NaCl). Inosine or sterile saline were administered between 9 a.m. and 11 a.m. before the CUMS procedures over the course of 4 weeks. All dose and pretreatment schedules were performed based on previous studies (Yuan et al., 2018), and the experimental design for the current study is shown in **Figure 1**.

### CUMS Model Paradigm

The CUMS paradigm was conducted using a previously published method, with minor modifications (Rao et al., 2016). Briefly, a series of unpredictable stressors were applied, including 1-min foot shock, 2-min tail clamp, 5-min swimming in 18°C, 1-h 4°C cold stress, 4-h restraint stress, 12-h stroboscopic illumination, 12-h inversion of dark/light cycle, 24-h paired housing, 24-h cage tilting at 45°, 24-h wet bedding cage, and 24-h food and water deprivation. Two of these stressors were performed every day for 4 weeks, and the same stressor was not applied over two consecutive days. Mice in the CON+saline group were handled with standard daily care.

### Behavioral Tests

#### SPT and Body Weight

The SPT was performed based on a previous study (Rao et al., 2016). After deprivation of food and water for 12 h before the test, mice were given a free choice between two bottles of liquid (200 ml 1% sucrose solution and 200 ml tap water), and the position of each bottle was exchanged after 3 h to avoid any side-preference effects. After 6 h, sucrose solution and tap water consumption were measured, and the sucrose preference rate was calculated using the equation: sucrose solution (g)/(sucrose solution (g) + water (g)) × 100%, and the body weights of all mice were assessed under the similar conditions weekly throughout the study.

#### LAT, OFT, and EPM

All mice were placed in a soundproof room to acclimatize for at least 15 min prior to the LAT, OFT, and EPM. Without stress stimulation, the LAT was performed to confirm the activity ability of mice. Mice were placed in the center of a chamber (50 × 50 × 40 cm), and the total distance traveled was quantified for 5 min *via* a computerized video tracking system (Ethovision XT 13, Noldus, Virginia, USA) in accordance with a previously published protocol (Yang et al., 2019). Total distance and the time spent in central squares were recorded as indexes of the OFT. The EPM test consists of an elevated, plus symbol-shaped apparatus with two open and two closed arms. Each mouse was placed in the center with its nose facing an open quadrant, and the times spent in open and closed quadrants were measured for 5 min. After each mouse finished the OFT and the EPM, the floor surfaces and walls were scrubbed with 75% alcohol to abolish any olfactory cues (Bai et al., 2021).

### Fecal Collection and 16S rRNA Sequencing

At the end of the study, fresh fecal samples were collected from all 75 mice and stored at −80°C. A total of 30 individual feces (10 individual feces in each group) were used in this study. In accordance with the manufacturer’s instructions, microbial genomic DNA was extracted from fecal samples using the E.Z.N.A.^®^ soil DNA Kit (Omega Bio-tek, Norcross, GA, USA). The V3–V4 region of the bacterial 16S rRNA gene was amplified with primer pairs 338F (5′-ACTCCTACGGGAGGCAGCAG-3′) and 806R (5′-GGACTACHVGGGTWTCTAAT-3′) and an ABI Gene Amp^®^ 9700 PCR thermocycler (ABI, Vernon, CA, USA). The PCR products were separated on agarose gels then sequenced *via* paired-end sequencing and an Illumina MiSeq sequencing platform (Illumina, San Diego, CA, USA) using PE300 (Majorbio Bio-Pharm Technology Co., Ltd., Shanghai, China) in accordance with the manufacturer’s protocol.

The resulting sequences were quality-filtered using fastp (0.19.6) (Chen S. et al., 2018) and merged *via* FLASH (v. 1.2.11) (Magoc and Salzberg, 2011). The high-quality sequences were then denoised using the DADA2 plugin (Callahan et al., 2016) in Qiime2 (v. 2020.2) (Bolyen et al., 2019) to obtain amplicon sequence variants (ASVs). Taxonomic assignment of ASVs was performed using the naive Bayes consensus taxonomy classifier implemented in Qiime2 and the SILVA 16S rRNA database (v. 138). Alpha diversity was calculated based on richness (Chao and ACE index) and diversity (Shannon and Simpson’s diversity index) to evaluate microbial community diversity, and principal coordinate analysis (PCoA) and partial least squares discriminant analysis (PLS-DA) were calculated to assess beta diversity. Linear discriminant analysis effect size (LEfSe) analysis of the 16S rRNA microbiome sequencing data was performed to identify discriminative bacterial taxa and KEGG categories among the groups, only when linear discriminant analysis (LDA) score >2.0 and *p*-value <0.05 were considered significant (http://huttenhower.sph.harvard.edu/galaxy) (Segata et al., 2011).

### Statistical Analysis

Data were expressed as means ± the standard error of the mean and were assessed using SPSS 21.0 (SPSS, Chicago, IL, USA) and Prism 8 (GraphPad, San Diego, CA, USA). Behavioral data were assessed for normality of distribution using the Shapiro-Wilk test. When the data exhibited a normal distribution and homogeneity of variance, one-way analysis of variance was used to assess behavioral tests. The nonparametric factorial Kruskal-Wallis *H*-test was used to compare nonnormally distributed variables. Correlations between microbial relative abundance and behavioral tests were assessed by Spearman’s correlational analysis. *p <*0.05 was deemed to indicate statistical significance.

## Results

### Effects of Inosine on CUMS-Induced Depressive and Anxiety-Like Behaviors

**Figure 2:**
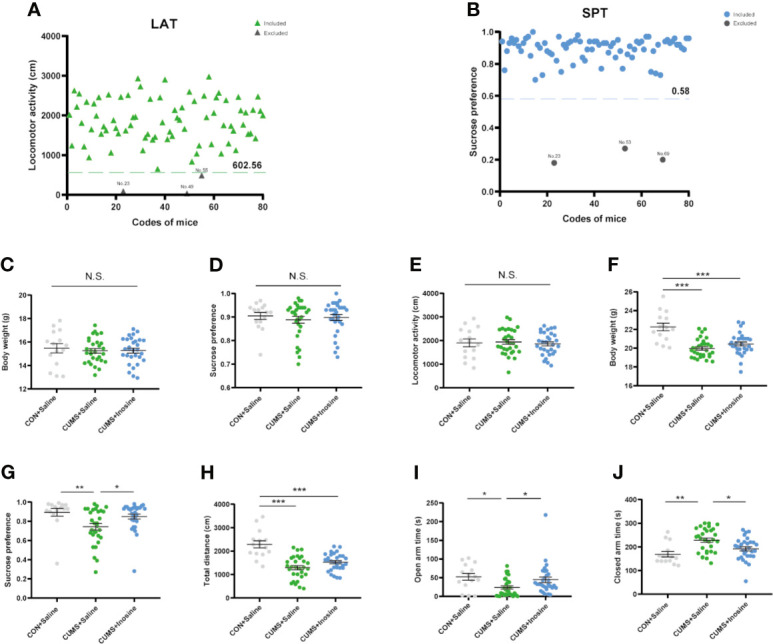
The exclusion criteria of baseline behavioral tests and results of behavioral tests at baseline and end of 4 weeks CUMS exposure. **(A)** The values below the 95% reference interval in the locomotor activity test (LAT). **(B)** The values below the 95% reference interval in the sucrose preference test (SPT). **(C)** The baseline test of body weight. **(D)** The baseline test of SPT. **(E)** The baseline test of LAT. **(F)** The endpoint of body weight. **(G)** The endpoint of SPT. **(H)** The endpoint total distance in open flied test. **(I)** The open-arm time in elevated plus maze (EPM). **(J)** The closed arm time in EPM. Data are presented as means ± SEM. Differences among the three groups were measured by one-way ANOVA and Kruskal-Wallis *H*-test. *p*-Value < 0.05 was considered statistically significant; *p < 0.05, **p < 0.01, ***p < 0.001—significant differences between two groups. ns, no significance.

The selection process and behaviors results are shown in **Figure 2**. Five mice were excluded after baseline LAT and SPT tests, and one of them was excluded due to both LAT and SPT. At baseline, there were no significant differences in body weight (*F*_(2, 72)_ = 0.181, *p* = 0.834), SPT (*F*_(2, 72)_ = 0.12, *p* = 0.942), or LAT (*F*_(2, 72)_ = 0.168, *p* = 0.845) among the three groups. At the end of the study, the body weights in the CUMS+inosine (*p* <0.001) and CUMS+saline (*p* <0.001) groups were significantly lower than CON+saline group. Compared with the CON+saline group, the CUMS+saline group exhibited significantly lower sucrose preference (*p* = 0.002), total distance in OFT (*p* <0.001), spending time in the open arm (*p* = 0.01), and higher spending time in the close arm (*p* = 0.001). In the CUMS+inosine group, inosine ameliorated the reductions in sucrose preference (*p* = 0.04) and time spent in the open arm (*p* = 0.029) and increased time spent in the closed arm (*p* = 0.038) compared with the CUMS+saline group.

### Inosine Treatment and the Modulation of Fecal Microbiota Composition

**Figure 3:**
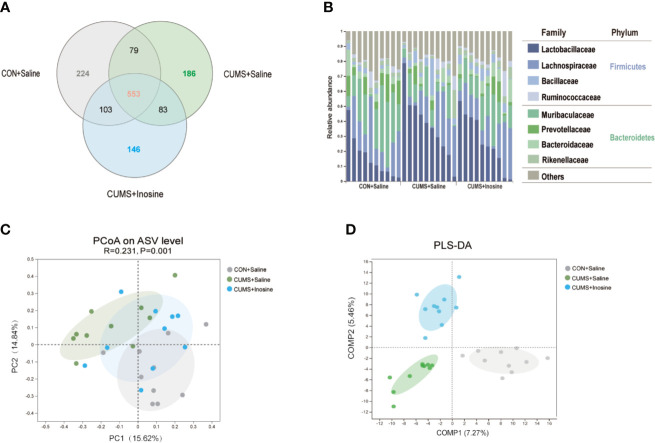
Comparison of gut microbiota composition among CON+saline, CUMS+saline, and CUMS+inosine groups. **(A)** Venn diagram showing ASV richness and the overlap of CON+saline (grey), CUMS+saline (green), and CUMS+inosine (blue) groups. **(B)** The relative abundance of ASVs assigned to family and phylum levels. **(C)** The principal coordinate analysis of samples along with principal components 1 and 2, which explained 14.84% and 15.62% of the total variance, respectively. **(D)** Partial least squares discriminant analysis plot of gut microbiota among three groups: CON+saline group (grey dots), CUMS+saline group (green dots), and CUMS+inosine group (blue dots).

A total of 838,226 high-quality reads were identified across all 30 samples. A Venn diagram indicated that 553 of the 1,374 ASVs were common to all three groups, and 224, 186, and 146 ASVs were respectively unique to the CON+saline, CUMS+saline, and CUMS+inosine groups (**Figure 3A**). The relative abundances of microbes at the phylum and family levels in the three groups are shown in **Figure 3B**. In all three groups, *Firmicutes* and *Bacteroidetes* were the dominant phyla in the fecal microbiota, and *Lactobacillaceae*, *Lachnospiraceae*, and *Muribaculaceae* were the most abundant families. In within-sample (α) phylogenetic diversity analysis of four indices (Ace, Shannon, Chao, and Simpson), there were no differences among the three groups (**Supplementary Figure S1**). At the ASV level, PCoA plot of Bray-Curtis indicated that the microbial composition was prominently discriminative among the three groups (*p =* 0.001, **Figure 3C**). Meanwhile, PLS-DA plot confirmed similar discrimination as well, and the fecal microbial composition of the three groups clustered separately (**Figure 3D**).

### Inosine Treatment Influenced Fecal Microbiota at Different Levels

**Figure 4:**
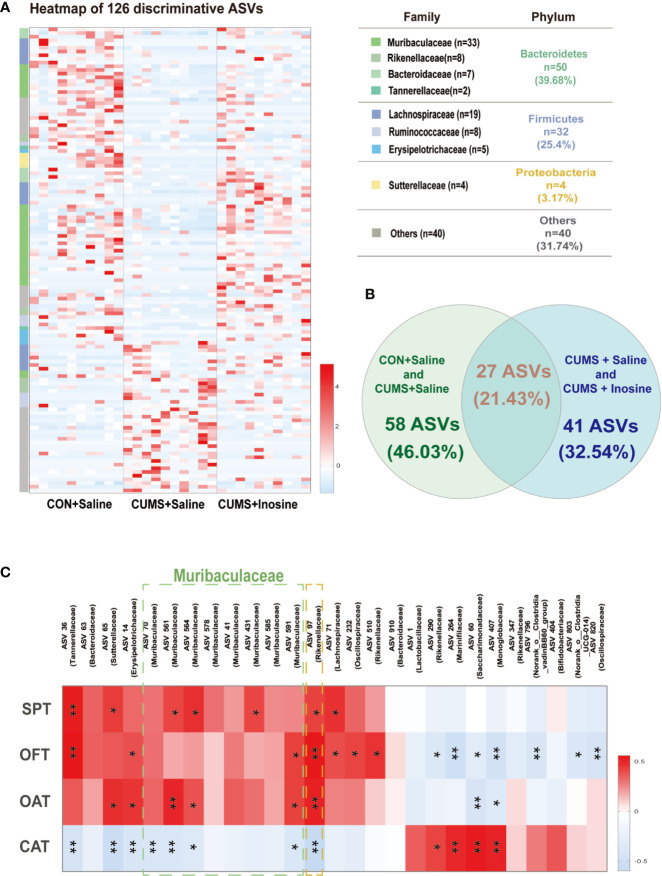
The discriminative ASVs among three groups and the association between discriminative ASVs and depressive and anxiety-like behaviors. **(A)** Heatmap of the 126 discriminative ASV abundances among the CON+saline, CUMS+saline, and CUMS+inosine groups (linear discriminant analysis (LDA) >2.0). ASVs (raw) were sorted by taxa and classified at family and phylum levels. The intensity of color in the middle heatmap (blue to red) indicated the normalized abundance score for each ASV. **(B)** Venn diagram for discriminative ASVs among the three groups. Green designates enriched different ASVs between the CON+saline and CUMS+saline groups; blue designates enriched discriminative ASVs between the CUMS+saline and CUMS+inosine groups. **(C)** Heatmap of Spearman’s rank the correlations between behavioral indices and bacterial abundance of mutual discriminative ASVs; *p < 0.05, **p < 0.01.

To determine distinct gut microbiota associated with inosine in detail, a LEfSe method was used to identify significantly discriminative gut microbiota ASVs in the CON+saline and CUMS+saline groups (**Supplementary Table S1**) and the CUMS+saline and CUMS+inosine groups (**Supplementary Table S2**). In total, 126 significantly altered ASVs were identified (**Figure 4A**), while 27 dramatically discrepant ASVs were shared in comparison with CON+saline and CUMS+saline groups and CUMS+saline and CUMS+inosine groups. Meanwhile, 58 ASVs were unique present in the CON+saline and CUMS+saline groups but absent in the CUMS+inosine group, and 41 ASVs were present in the CUMS+saline and CUMS+inosine groups but absent in the CON+saline group (**Figure 4B**). At the phylum level, the majority of discriminative ASVs originated from *Bacteroidetes* (39.68%) and *Firmicutes* (25.40%). At the family level, large proportions of different ASVs belonged to *Muribaculaceae* (26.19%) and *Lachnospiraceae* (15.08%). Interestingly, 27 common discrepant ASVs were altered after CUMS stimulation and recovered by inosine. The majority of mutual differential ASVs (19/27, 70.37%), mainly belonging to *Muribaculaceae* (5/19, 26.32%), were correlated with at least one depressive or anxiety-like behavioral phenotype including SPT, OFT, open arm time, and closed arm time. Only ASV97 originating from the family *Rikenellaceae* was significantly associated with all behavioral tests (**Figure 4C**).

### Fecal Microbiome and Alteration of Related Functional Signaling Pathways

**Figure 5:**
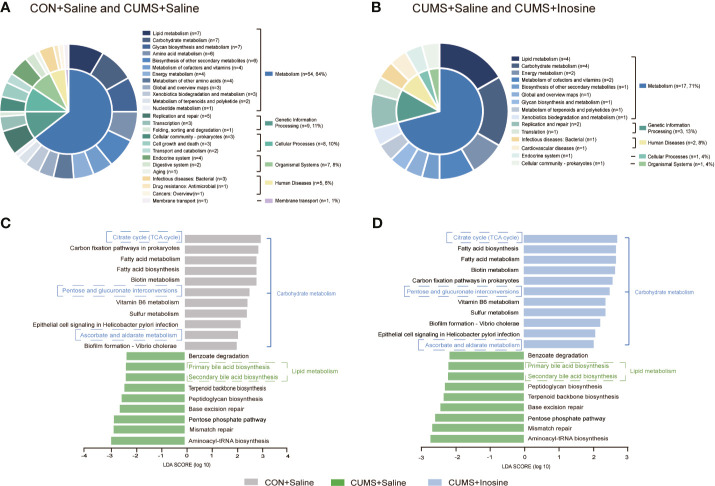
The related functional enrichment in the KEGG pathway. **(A)** The pie chart of discriminative KEGG pathways between the CON+saline and CUMS+saline groups. **(B)** The pie chart of discriminative KEGG pathways between the CUMS+saline and CUMS+inosine groups. **(C)** The mutual discriminative KEGG pathways among three groups representing in CON+saline and CUMS+saline groups. **(D)** The mutual discriminative KEGG pathways among three groups representing in CUMS+saline and CUMS+inosine groups. The discriminative KEGG pathways among three groups were identified by linear discriminant analysis effect size, only when LDA > 2.0 and *p*-value < 0.05 were considered significant.

To identify the functions of the key bacterial taxon, LEfSe analysis was used to assess the KEGG pathway (LDA >2.0 and *p* <0.05) in the three groups. Functional enrichment identified 84 KEGG pathways for discriminating the fecal microbiomes in the CON+saline and CUMS+saline groups (**Figure 5A**) and 24 for discriminating the fecal microbiomes in the CUMS+saline and CUMS+inosine groups (**Figure 5B**). These differential pathways were mainly associated with metabolism, including 64.29% in the CON+saline and CUMS+saline groups and 70.83% in the CUMS+saline and CUMS+inosine groups. Lipid metabolism (7/84, 8.33%), glycan biosynthesis and metabolism (7/84, 8.33%), and carbohydrate metabolism (7/84, 8.33%) were dominant in the CON+saline and CUMS+saline groups, whereas lipid metabolism (4/24, 16.67%) and carbohydrate metabolism (4/24, 16.67%) were dominant in the CUMS+saline and CUMS+inosine groups. In enrichment analysis, there were 20 significantly altered pathways in comparisons between the CON+saline and CUMS+saline groups and the CUMS+saline and CUMS+inosine groups (**Figures 5C, D** **)**. Compared with the CUMS+saline group, carbohydrate metabolism (citrate cycle, pentose and glucuronate interconversions, and ascorbate and aldarate metabolism) was significantly more abundant in both the CON+saline and CUMS+inosine groups. Meanwhile, lipid metabolism (primary and secondary bile acid metabolism) was significantly more abundant in the CUMS+saline group. These findings suggest that inosine might affect the gut microbiome *via* carbohydrate metabolism and lipid metabolism pathways.

## Discussion

Growing evidence suggests a relationship between gut microbiota and antidepressants (Burokas et al., 2017), but the specific mechanisms involved are unclear (Park et al., 2018). Herein, we reported that young CUMS mice were characterized by depressive and anxiety-like behaviors and gut microbiota dysbiosis. Inosine could significantly ameliorate depressive and anxiety-like behaviors and was associated with partial restoration of gut microbiota dysbiosis induced by CUMS stimulations. A majority of discriminative ASVs belonging to the *Muribaculaceae* family were strongly positively associated with depressive behaviors. The altered microbial compositions observed were linked with dysregulation of carbohydrate metabolism and lipid metabolism. To our knowledge, this is the first study indicating that inosine improves depressive and anxiety-like behaviors in association with the alteration of gut microbiota in adolescent mice, and it may provide a fresh perspective on the antidepressant effects of inosine.

Depression in children and adolescents is viewed as a special classification. A comprehensive meta-analysis indicated that pharmacological therapy in children and adolescents with MDD yielded results that were far from satisfactory and suggested that venlafaxine might increase the significant risk of suicidality (suicidal behavior or ideation) in young people (Cipriani et al., 2016). This might be explained as follows. The nervous system and receptor mechanisms are undeveloped in children and young adolescents (Bylund and Reed, 2007), it may intensify emotional feedback to negative social stimuli *via* dysregulation of the hypothalamic-pituitary-adrenal axis (Cousins and Goodyer, 2015). Moreover, choosing an effective concentration in young patients of different ages is complex, particularly given the differences in pharmacokinetic properties of antidepressants (Kozisek et al., 2007). Kaster et al. (2013) reported specific antidepressant-like effects of inosine in mice subjected to the forced swimming test and the tail suspension test. The brain levels of inosine increased after oral administration and prevented chronic social defeat stress-induced reduction of performance in the SPT in adult mice (Muto et al., 2014). The present study investigated whether inosine could significantly improve depressive and anxiety-like behaviors in young CUMS mice.

In the present study, the majority of discriminative ASVs belonged to the *Firmicutes* and *Bacteroidetes* phyla, which is concordant with previous studies. Jiang et al. (2015) reported that *Firmicutes* was significantly reduced compared with the healthy control group, whereas *Bacteroidetes* had strongly increased levels in the MDD groups. This is consistent with Huang et al. (2018) in which *Firmicutes* and *Bacteroidetes* were the two most abundant phyla in healthy control and MDD groups, and patients with MDD had significantly less *Firmicutes*. There is also emerging evidence that the Firmicutes/Bacteroidetes ratio is closely connected with gut dysbiosis (Grigor’eva, 2020), and Firmicutes have been associated with immune-mediated inflammatory and metabolic diseases such as irritable bowel syndrome (Jeffery et al., 2012) and obesity (Turnbaugh et al., 2006). In the current study, the majority of differential ASVs belonged to the *Muribaculaceae* family, and there was a significant positive correlation between depressive and anxiety-like behaviors and the abundance of *Muribaculaceae*. The abundance of *Muribaculaceae* microbes belonging to the Bacteroidetes phylum was reportedly strongly associated with metabolizing carbohydrates (Chung et al., 2020), polyphenols (Selma et al., 2017), and propionate (Smith et al., 2019). It has been reported that *Muribaculaceae* play a cooperative role in the production of short-chain fatty acids (SCFAs) (Smith et al., 2019). SCFAs could stimulate the vagus nerve (Bonaz et al., 2019), induce enteroendocrine cells to produce neuropeptides, activate afferent nerve pathways (Sarkar et al., 2016), and participate in local neurotransmitter production and systemic regulation *via* tryptophan metabolism or direct secretion (O’Mahony et al., 2015; Yano et al., 2015). Potent anti-inflammatory properties have also been reported in this context (Baxter et al., 2019). *Muribaculaceae* and associated coproduced short-chain fatty acids have been significantly correlated with depression (Duan et al., 2021), but there is no direct evidence to prove the relevant brain mechanism in MDD. Currently, inosine is commonly used in the treatment of Parkinson’s disease (Schwarzschild et al., 2014) and multiple sclerosis (Markowitz et al., 2009), and it has exhibited direct anti-inflammatory effects on pleural inflammation (da Rocha Lapa et al., 2012). It has been widely reported that inflammatory responses are significantly increased in patients with MDD (Miller et al., 2009), and we proposed that exogenous inosine supplementation may affect inflammation-related metabolites associated with gut microbiota such as short-chain fatty acids.

Although the specific mechanisms by which the gut microbiome may affect the brain to modulate depressive behavior are unclear, the results of the current study are consistent with reports that MDD is usually accompanied by disturbances of lipid metabolism (Zheng et al., 2012; Jia et al., 2016; Pu et al., 2020a). Lipid metabolism was enriched in the CUMS+saline group compared with the CON+saline group and the CUMS+inosine group, suggesting that CUMS stress caused the lipid metabolism disturbances, and that they may be partially reversed by inosine. Yuan et al. (2020) reported that genetic polymorphisms associated with lipid metabolism systems influenced antidepressant response in MDD patients. In MDD, disturbances of lipid metabolism are closely associated with monoamine transmitters (Zhou et al., 2020), brain-derived neurotrophic factor (BDNF) (Phillips, 2017), and the immune system (Dantzer et al., 2008). It is well established that monoamine neurotransmitters such as 5-hydroxytryptamine (5-HT) in the central nervous system play a key role in the pathophysiology of depression (Jans et al., 2007). Zhou et al. (2020) reported that disturbances in lipid metabolism affected 5-HT reabsorption in the central nervous system. Depressive symptoms have also been associated with reduced BDNF synthesis, indicating that decreased BDNF levels increased vulnerability to stress and depression (Duman and Monteggia, 2006). Moreover, carbohydrate metabolism differed significantly in three groups (Pu et al., 2020b). As mentioned above, *Muribaculaceae* have exhibited a close association with carbohydrates (Chung et al., 2020), and *Firmicutes* and *Bacteroidetes* are regarded as crucial components in the regulation of carbohydrate metabolism involving gut microbes (Cuskin et al., 2015; Sheridan et al., 2016). Although we did not determine whether the effects of inosine on gut microbiota dysfunction were due to modulation of these bacterial phyla, the study provided further scientific evidence that gut microbiota is an important factor associated with the effects of inosine.

The current study had some limitations. (1) Although CUMS stimulation in mice is widely accepted as a classic depressive animal model, it is not representative of the complex characteristics of human patients with MDD. Other animal models and clinical studies are needed to elucidate gut microbiota mechanisms associated with inosine. (2) We have conducted preliminary experiments investigating the effects of inosine in normal adolescent rats without stress stimulation (i.e., Control + inosine rats), and inosine had antidepressant-like effects as evidenced by the forced swimming test (Yuan et al., 2018). Nevertheless, the CON+inosine group should be set to further determine the role of inosine in this study. (3) The gut microbiota observed in the present study may be a target of inosine, thus affecting depressive and anxiety-like behaviors. Notably however, it is not known whether the altered gut microbiota observed in the current study is a crucial factor or an accompanying consequence of stress exposure and inosine treatment. Thus, we still consider that fecal microbiota transplantation is essential to verify the analysis of 16S rRNA sequencing results in the present study. (4) To avoid hormone fluctuation leading to a dynamic neuroendocrine environment in female mice, which influences neuron structure, neural reward function, and motivation, and is related to neuropsychiatric disorders such as depression (Oyola and Handa, 2017), only male mice were included in the current study (Paden et al., 2020). However, sexual dimorphism is reportedly associated with clinical treatment differences and differences in gut microbiota between female and male MDD patients (Labonte et al., 2017; Chen J. J. et al., 2018). The present study should also be conducted in female C57BL/6 mice to assess potential effects of a sex bias. (5) The limited sample sizes in the study may restrict the interpretation of results.

In conclusion, antidepressant and antianxiety effects of inosine were evident in young CUMS mice in the present study and were accompanied by alterations in gut microbiota and metabolic pathways. Inosine affected fecal microbiota composition in CUMS mice, and 126 discriminative ASVs were identified in the three groups, including 27 shared altering discriminative ASVs. Most of the discriminative ASVs belonged to the *Bacteroidetes* and *Firmicutes* phyla and the *Muribaculaceae* and *Lachnospiraceae* families. The *Muribaculaceae* family was significantly positively correlated with depressive and anxiety-like behaviors. Lipid and carbohydrate metabolism were identified as two potentially vital metabolic pathways involved in the interaction between inosine and the gut microbiome. Collectively, the results of the current study provide a novel perspective on the antidepressant effects of inosine.

## Data Availability Statement

The data presented in the study are deposited in the NCBI repository, accession number PRJNA749870.

## Ethics Statement

The animal study was reviewed and approved by the Animal Ethics Committee of Chongqing Medical University.

## Author Contributions

PX and XZ designed the experiments. XLiu, TT, XLi, LF, YX, YJ, KD, and YZ conducted the experiments, XLiu, TT, XZ, and PX analyzed the data and drafted the manuscript. All authors contributed to the article and approved the submitted version.

## Funding

This research was supported by the National Basic Research Program of China (Grant No. 2017YFA0505700 to PX), the Nonprofit Central Research Institute Fund of Chinese Academy of Medical Sciences (Grant No. 2019PT320002 to PX), the projects of International Cooperation and Exchanges NSFC (Grant No. 81820108015 to PX), the National Natural Science Foundation of China (Grant No. 81701342 to XZ), the Natural Science Foundation of China (Grant No. 82001434 to YZ), and the Natural Science Foundation of Chongqing (cstc2020jcyjmsxmX0149 to YZ).

## Conflict of Interest

The authors declare that the research was conducted in the absence of any commercial or financial relationships that could be construed as a potential conflict of interest.

## Publisher’s Note

All claims expressed in this article are solely those of the authors and do not necessarily represent those of their affiliated organizations, or those of the publisher, the editors and the reviewers. Any product that may be evaluated in this article, or claim that may be made by its manufacturer, is not guaranteed or endorsed by the publisher.

## Supplementary Material

Supplementary Figure S1 Gut microbial characteristics of adolescent CUMS mice at the α-diversity analysis. **(A)** The ACE index of α-diversity analysis among three groups. **(B)** The Chao index of α-diversity analysis among three groups. **(C)** The Shannon index of α-diversity analysis among three groups. **(D)** The Simpson index of α-diversity analysis among three groups.

Supplementary Figure S2 The results of body weight and sucrose preference from baseline to endpoint. **(A)** The body weight. **(B)** The sucrose preference.
