# Supplementary figures and images for: Impact of Inosine on Chronic Unpredictable Mild Stress-Induced Depressive and Anxiety-Like Behaviors With the Alteration of Gut Microbiota
Source: Front Cell Infect Microbiol. 2021 Sep 14;11:697640. doi: 10.3389/fcimb.2021.697640 (PMC8476956; doi:10.3389/fcimb.2021.697640)

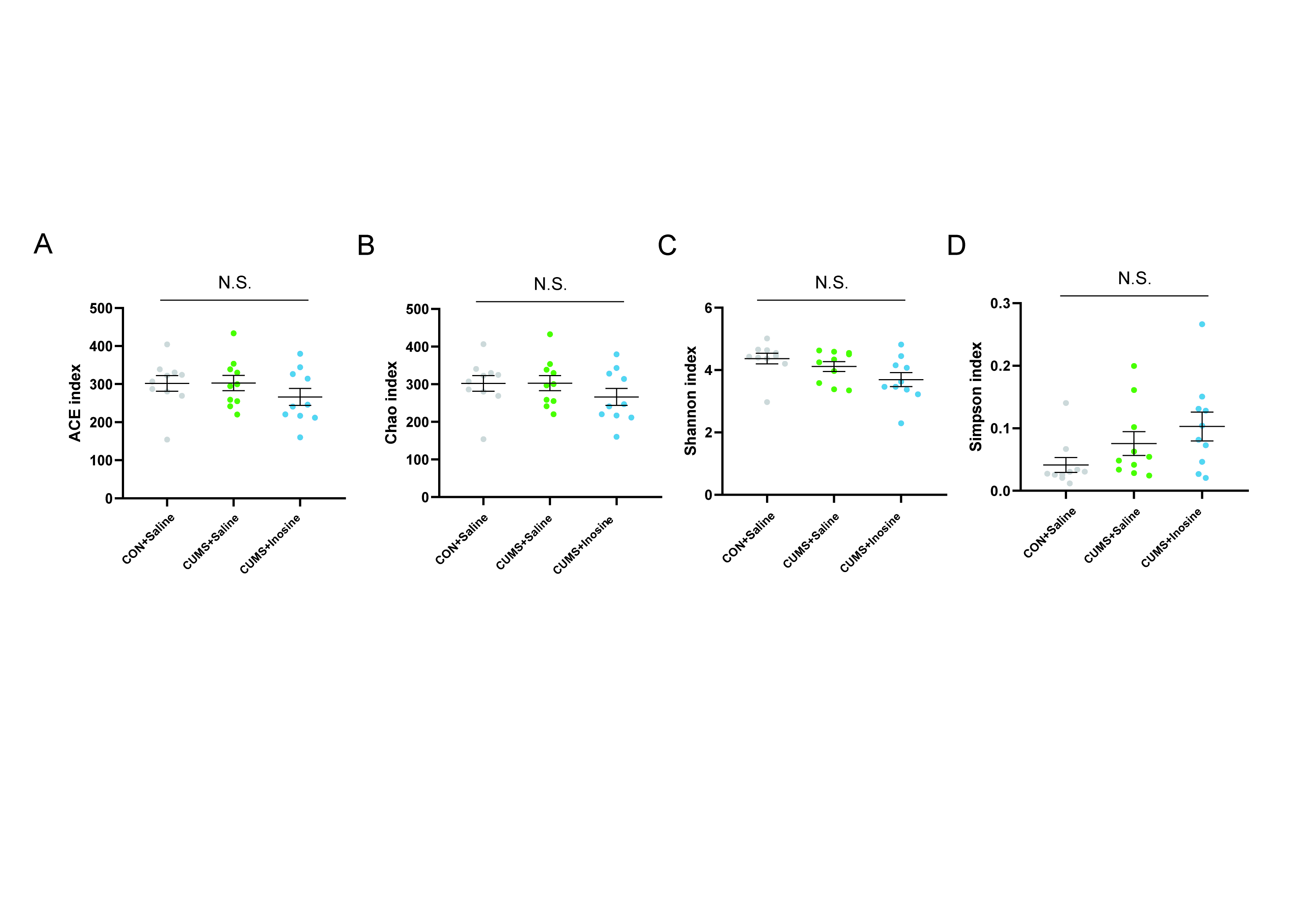

Supplement: Supplementary Figure S1 — Gut microbial characteristics of adolescent CUMS mice at the α-diversity analysis. (A) The ACE index of α-diversity analysis among three groups. (B) The Chao index of α-diversity analysis among three groups. (C) The Shannon index of α-diversity analysis among three groups. (D) The Simpson index of α-diversity analysis among three groups. [file Image_1.tif]

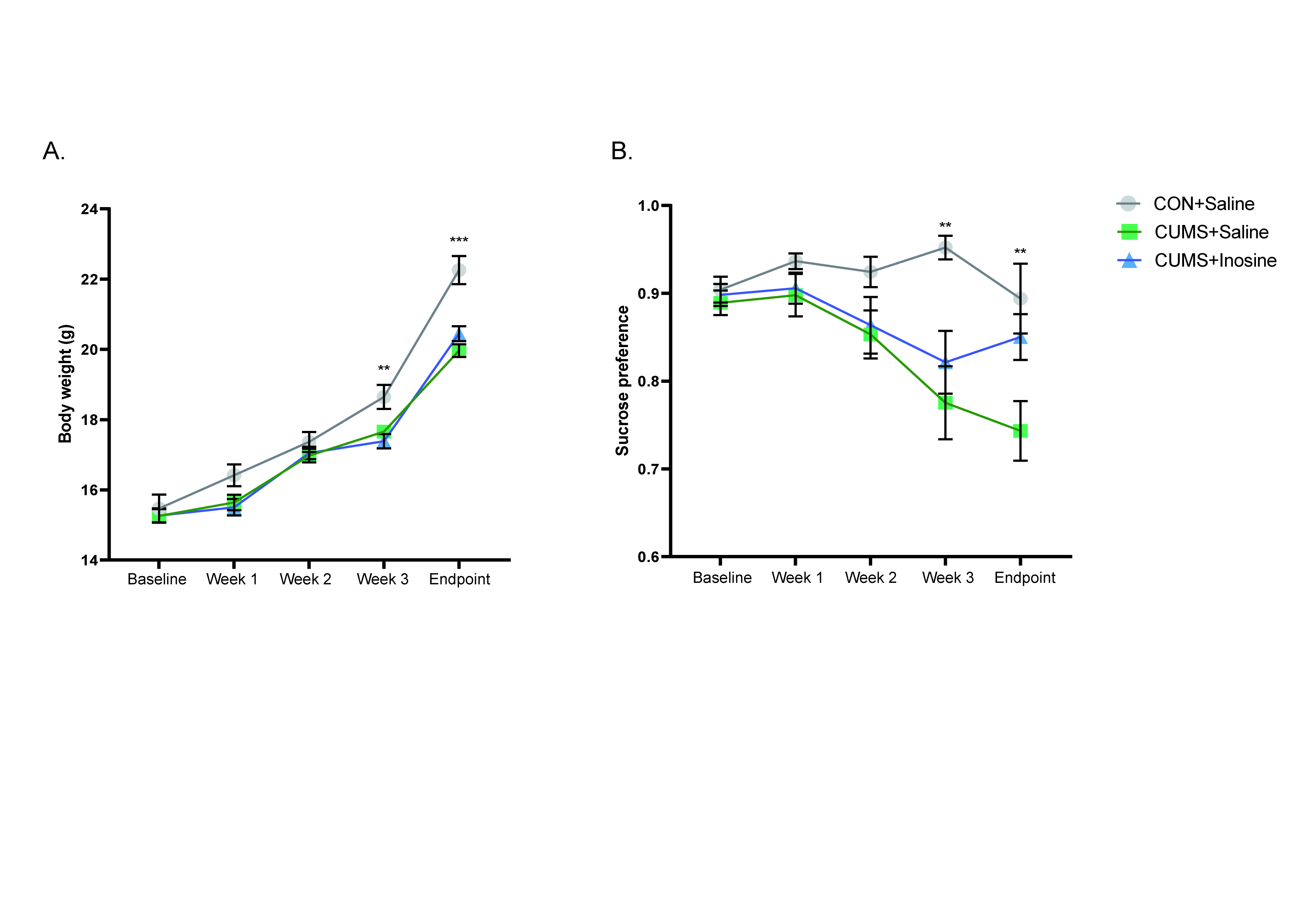

Supplement: Supplementary Figure S2 — The results of body weight and sucrose preference from baseline to endpoint. (A) The body weight. (B) The sucrose preference. [file Image_2.tif]
