# Supplementary material for: Impact of Inosine on Chronic Unpredictable Mild Stress-Induced Depressive and Anxiety-Like Behaviors With the Alteration of Gut Microbiota
Source: Front Cell Infect Microbiol. 2021 Sep 14;11:697640. doi: 10.3389/fcimb.2021.697640 (PMC8476956; doi:10.3389/fcimb.2021.697640)
Supplement: Supplementary file 3 [file Table_1.docx]

**Table S1.** The 85 discriminative ASVs between CON+Saline and CUMS+Saline groups identified by LEfSe.

| **Species name** | **Family** | **Phylum** | **LDA value** | **P value** |
| --- | --- | --- | --- | --- |
| **ASV1** | Lactobacillaceae | Firmicutes | 4.832 | 0.013 |
| **ASV5** | Lactobacillaceae | Firmicutes | 4.502 | 0.009 |
| **ASV6** | Bacillaceae | Firmicutes | 4.286 | 0.013 |
| **ASV70** | Muribaculaceae | Bacteroidota | 4.093 | 0.011 |
| **ASV7** | Muribaculaceae | Bacteroidota | 3.857 | 0.010 |
| **ASV290** | Rikenellaceae | Bacteroidota | 3.854 | 0.009 |
| **ASV51** | Muribaculaceae | Bacteroidota | 3.785 | 0.039 |
| **ASV264** | Marinifilaceae | Bacteroidota | 3.779 | 0.001 |
| **ASV10** | Bacteroidaceae | Bacteroidota | 3.703 | 0.003 |
| **ASV455** | Prevotellaceae | Bacteroidota | 3.656 | 0.022 |
| **ASV55** | Unclassified_o_Oscillospirales | Firmicutes | 3.622 | 0.033 |
| **ASV456** | Lachnospiraceae | Firmicutes | 3.593 | 0.027 |
| **ASV19** | Muribaculaceae | Bacteroidota | 3.590 | 0.005 |
| **ASV36** | Tannerellaceae | Bacteroidota | 3.514 | 0.000 |
| **ASV12** | Muribaculaceae | Bacteroidota | 3.500 | 0.018 |
| **ASV28** | Muribaculaceae | Bacteroidota | 3.427 | 0.015 |
| **ASV277** | Rikenellaceae | Bacteroidota | 3.390 | 0.018 |
| **ASV300** | Muribaculaceae | Bacteroidota | 3.304 | 0.031 |
| **ASV60** | Saccharimonadaceae | Patescibacteria | 3.264 | 0.005 |
| **ASV63** | Bacteroidaceae | Bacteroidota | 3.227 | 0.031 |
| **ASV561** | Muribaculaceae | Bacteroidota | 3.223 | 0.017 |
| **ASV229** | Erysipelotrichaceae | Firmicutes | 3.212 | 0.041 |
| **ASV82** | Lachnospiraceae | Firmicutes | 3.201 | 0.033 |
| **ASV65** | Sutterellaceae | Proteobacteria | 3.196 | 0.002 |
| **ASV14** | Erysipelotrichaceae | Firmicutes | 3.194 | 0.015 |
| **ASV601** | Norank_o_Clostridia_UCG-014 | Firmicutes | 3.184 | 0.031 |
| **ASV244** | Selenomonadaceae | Firmicutes | 3.142 | 0.002 |
| **ASV1059** | Lachnospiraceae | Firmicutes | 3.138 | 0.031 |
| **ASV564** | Muribaculaceae | Bacteroidota | 3.122 | 0.019 |
| **ASV278** | Lachnospiraceae | Firmicutes | 3.120 | 0.013 |
| **ASV274** | Marinifilaceae | Bacteroidota | 3.100 | 0.041 |
| **ASV578** | Muribaculaceae | Bacteroidota | 3.072 | 0.031 |
| **ASV41** | Muribaculaceae | Bacteroidota | 3.050 | 0.038 |
| **ASV324** | Norank_o_Clostridia_vadinBB60_group | Firmicutes | 3.049 | 0.031 |
| **ASV111** | Muribaculaceae | Bacteroidota | 3.027 | 0.022 |
| **ASV518** | Eggerthellaceae | Actinobacteriota | 3.021 | 0.021 |
| **ASV416** | Ruminococcaceae | Firmicutes | 3.021 | 0.031 |
| **ASV440** | norank_o__Clostridia_UCG-014 | Firmicutes | 3.002 | 0.013 |
| **ASV101** | Ruminococcaceae | Firmicutes | 2.980 | 0.023 |
| **ASV87** | Muribaculaceae | Bacteroidota | 2.967 | 0.027 |
| **ASV307** | Rikenellaceae | Bacteroidota | 2.945 | 0.019 |
| **ASV407** | Monoglobaceae | Firmicutes | 2.934 | 0.002 |
| **ASV347** | Rikenellaceae | Bacteroidota | 2.909 | 0.013 |
| **ASV538** | Norank_o__Clostridia_vadinBB60_group | Firmicutes | 2.904 | 0.031 |
| **ASV331** | Lachnospiraceae | Firmicutes | 2.901 | 0.026 |
| **ASV172** | Rikenellaceae | Bacteroidota | 2.863 | 0.017 |
| **ASV373** | Norank_o__Clostridia_UCG-014 | Firmicutes | 2.859 | 0.025 |
| **ASV98** | Sutterellaceae | Proteobacteria | 2.856 | 0.017 |
| **ASV343** | Lachnospiraceae | Firmicutes | 2.855 | 0.026 |
| **ASV279** | Lachnospiraceae | Firmicutes | 2.854 | 0.042 |
| **ASV97** | Rikenellaceae | Bacteroidota | 2.846 | 0.001 |
| **ASV431** | Muribaculaceae | Bacteroidota | 2.842 | 0.011 |
| **ASV585** | Muribaculaceae | Bacteroidota | 2.838 | 0.013 |
| **ASV311** | Lachnospiraceae | Firmicutes | 2.832 | 0.012 |
| **ASV370** | Ruminococcaceae | Firmicutes | 2.816 | 0.031 |
| **ASV796** | Norank_o__Clostridia_vadinBB60_group | Firmicutes | 2.811 | 0.013 |
| **ASV569** | Muribaculaceae | Bacteroidota | 2.808 | 0.025 |
| **ASV516** | Tannerellaceae | Bacteroidota | 2.804 | 0.050 |
| **ASV404** | Bifidobacteriaceae | Actinobacteriota | 2.802 | 0.031 |
| **ASV742** | Saccharimonadaceae | Patescibacteria | 2.792 | 0.050 |
| **ASV91** | Unclassified_c__Bacilli | Firmicutes | 2.781 | 0.036 |
| **ASV308** | Saccharimonadaceae | Patescibacteria | 2.780 | 0.036 |
| **ASV69** | Anaerovoracaceae | Firmicutes | 2.774 | 0.004 |
| **ASV128** | Rikenellaceae | Bacteroidota | 2.765 | 0.008 |
| **ASV72** | Bacteroidaceae | Bacteroidota | 2.758 | 0.028 |
| **ASV803** | Norank_o__Clostridia_UCG-014 | Firmicutes | 2.749 | 0.031 |
| **ASV820** | Oscillospiraceae | Firmicutes | 2.748 | 0.031 |
| **ASV71** | Lachnospiraceae | Firmicutes | 2.747 | 0.027 |
| **ASV75** | Lachnospiraceae | Firmicutes | 2.728 | 0.009 |
| **ASV688** | Muribaculaceae | Bacteroidota | 2.712 | 0.031 |
| **ASV336** | Staphylococcaceae | Firmicutes | 2.707 | 0.031 |
| **ASV131** | Sutterellaceae | Proteobacteria | 2.702 | 0.031 |
| **ASV88** | Lachnospiraceae | Firmicutes | 2.697 | 0.041 |
| **ASV186** | Norank_o__Clostridia_vadinBB60_group | Firmicutes | 2.693 | 0.036 |
| **ASV209** | Sutterellaceae | Proteobacteria | 2.692 | 0.021 |
| **ASV232** | Oscillospiraceae | Firmicutes | 2.690 | 0.031 |
| **ASV851** | Oscillospiraceae | Firmicutes | 2.686 | 0.031 |
| **ASV510** | Rikenellaceae | Bacteroidota | 2.682 | 0.041 |
| **ASV157** | Norank_o__Clostridia_UCG-014 | Firmicutes | 2.660 | 0.031 |
| **ASV910** | Bacteroidaceae | Bacteroidota | 2.653 | 0.031 |
| **ASV1058** | Lachnospiraceae | Firmicutes | 2.639 | 0.031 |
| **ASV458** | Ruminococcaceae | Firmicutes | 2.605 | 0.041 |
| **ASV591** | Muribaculaceae | Bacteroidota | 2.591 | 0.007 |
| **ASV766** | Bacteroidaceae | Bacteroidota | 2.563 | 0.031 |
| **ASV223** | Lachnospiraceae | Firmicutes | 2.539 | 0.039 |

Red: increased in CUMS+Saline group; Green: decreased in CUMS+Saline group;
