# Supplementary material for: Impact of Inosine on Chronic Unpredictable Mild Stress-Induced Depressive and Anxiety-Like Behaviors With the Alteration of Gut Microbiota
Source: Front Cell Infect Microbiol. 2021 Sep 14;11:697640. doi: 10.3389/fcimb.2021.697640 (PMC8476956; doi:10.3389/fcimb.2021.697640)
Supplement: Supplementary file 4 [file Table_2.docx]

**Table S2.** The 68 discriminative ASVs between CUMS+Saline and CUMS+Inosine groups identified by LEfSe.

| **Species name** | **Family** | **Phylum** | **LDA value** | **P value** |
| --- | --- | --- | --- | --- |
| **ASV1** | Lactobacillaceae | Firmicutes | 4.715 | 0.049 |
| **ASV290** | Rikenellaceae | Bacteroidota | 3.922 | 0.049 |
| **ASV264** | Marinifilaceae | Bacteroidota | 3.710 | 0.010 |
| **ASV53** | Muribaculaceae | Bacteroidota | 3.410 | 0.023 |
| **ASV70** | Muribaculaceae | Bacteroidota | 3.384 | 0.024 |
| **ASV67** | Ruminococcaceae | Firmicutes | 3.379 | 0.041 |
| **ASV63** | Bacteroidaceae | Bacteroidota | 3.354 | 0.025 |
| **ASV78** | Ruminococcaceae | Firmicutes | 3.319 | 0.023 |
| **ASV95** | Lachnospiraceae | Firmicutes | 3.282 | 0.022 |
| **ASV359** | Lachnospiraceae | Firmicutes | 3.229 | 0.015 |
| **ASV825** | Norank_o__Gastranaerophilales | Cyanobacteria | 3.211 | 0.031 |
| **ASV60** | Saccharimonadaceae | Patescibacteria | 3.172 | 0.023 |
| **ASV41** | Muribaculaceae | Bacteroidota | 3.133 | 0.001 |
| **ASV301** | Muribaculaceae | Bacteroidota | 3.118 | 0.012 |
| **ASV453** | Norank_o__RF39 | Firmicutes | 3.114 | 0.031 |
| **ASV76** | Muribaculaceae | Bacteroidota | 3.104 | 0.031 |
| **ASV106** | Erysipelotrichaceae | Firmicutes | 3.100 | 0.041 |
| **ASV871** | Muribaculaceae | Bacteroidota | 3.098 | 0.013 |
| **ASV36** | Tannerellaceae | Bacteroidota | 3.088 | 0.005 |
| **ASV785** | Muribaculaceae | Bacteroidota | 3.087 | 0.005 |
| **ASV564** | Muribaculaceae | Bacteroidota | 3.070 | 0.009 |
| **ASV195** | Prevotellaceae | Bacteroidota | 3.046 | 0.049 |
| **ASV97** | Rikenellaceae | Bacteroidota | 3.038 | 0.026 |
| **ASV71** | Lachnospiraceae | Firmicutes | 3.035 | 0.002 |
| **ASV556** | Muribaculaceae | Bacteroidota | 3.002 | 0.020 |
| **ASV328** | Muribaculaceae | Bacteroidota | 2.994 | 0.019 |
| **ASV38** | Muribaculaceae | Bacteroidota | 2.981 | 0.028 |
| **ASV14** | Erysipelotrichaceae | Firmicutes | 2.980 | 0.025 |
| **ASV323** | Lachnospiraceae | Firmicutes | 2.974 | 0.027 |
| **ASV333** | Bacteroidaceae | Bacteroidota | 2.950 | 0.022 |
| **ASV184** | Lachnospiraceae | Firmicutes | 2.950 | 0.045 |
| **ASV820** | Oscillospiraceae | Firmicutes | 2.941 | 0.031 |
| **ASV232** | Oscillospiraceae | Firmicutes | 2.928 | 0.039 |
| **ASV397** | Oscillospiraceae | Firmicutes | 2.902 | 0.013 |
| **ASV431** | Muribaculaceae | Bacteroidota | 2.898 | 0.011 |
| **ASV43** | Muribaculaceae | Bacteroidota | 2.880 | 0.011 |
| **ASV796** | Norank_o__Clostridia_vadinBB60_group | Firmicutes | 2.866 | 0.013 |
| **ASV756** | Erysipelotrichaceae | Firmicutes | 2.823 | 0.031 |
| **ASV426** | Bacteroidaceae | Bacteroidota | 2.813 | 0.050 |
| **ASV379** | Norank_o__Clostridia_vadinBB60_group | Firmicutes | 2.812 | 0.036 |
| **ASV605** | Ruminococcaceae | Firmicutes | 2.800 | 0.041 |
| **ASV347** | Rikenellaceae | Bacteroidota | 2.787 | 0.041 |
| **ASV378** | Lachnospiraceae | Firmicutes | 2.785 | 0.026 |
| **ASV407** | Monoglobaceae | Firmicutes | 2.783 | 0.019 |
| **ASV561** | Muribaculaceae | Bacteroidota | 2.782 | 0.005 |
| **ASV775** | Lachnospiraceae | Firmicutes | 2.779 | 0.002 |
| **ASV391** | Norank_o__Clostridia_UCG-014 | Firmicutes | 2.777 | 0.013 |
| **ASV65** | Sutterellaceae | Proteobacteria | 2.776 | 0.048 |
| **ASV119** | Muribaculaceae | Bacteroidota | 2.761 | 0.026 |
| **ASV591** | Muribaculaceae | Bacteroidota | 2.754 | 0.009 |
| **ASV773** | Muribaculaceae | Bacteroidota | 2.752 | 0.033 |
| **ASV218** | Erysipelotrichaceae | Firmicutes | 2.747 | 0.041 |
| **ASV396** | Streptococcaceae | Firmicutes | 2.745 | 0.015 |
| **ASV149** | Oscillospiraceae | Firmicutes | 2.738 | 0.027 |
| **ASV757** | Muribaculaceae | Bacteroidota | 2.725 | 0.036 |
| **ASV510** | Rikenellaceae | Bacteroidota | 2.722 | 0.041 |
| **ASV771** | Muribaculaceae | Bacteroidota | 2.715 | 0.013 |
| **ASV549** | Prevotellaceae | Bacteroidota | 2.691 | 0.013 |
| **ASV588** | Muribaculaceae | Bacteroidota | 2.685 | 0.048 |
| **ASV803** | Norank_o__Clostridia_UCG-014 | Firmicutes | 2.678 | 0.031 |
| **ASV754** | Ruminococcaceae | Firmicutes | 2.676 | 0.005 |
| **ASV404** | Bifidobacteriaceae | Actinobacteriota | 2.658 | 0.031 |
| **ASV781** | Christensenellaceae | Firmicutes | 2.633 | 0.026 |
| **ASV585** | Muribaculaceae | Bacteroidota | 2.607 | 0.031 |
| **ASV578** | Muribaculaceae | Bacteroidota | 2.598 | 0.031 |
| **ASV910** | Bacteroidaceae | Bacteroidota | 2.594 | 0.013 |
| **ASV891** | Lachnospiraceae | Firmicutes | 2.584 | 0.031 |
| **ASV562** | Muribaculaceae | Bacteroidota | 2.550 | 0.047 |

Red: increased in CUMS+Saline group; Green: decreased in CUMS+Saline group;
