# Supplementary material for: Impact of Inosine on Chronic Unpredictable Mild Stress-Induced Depressive and Anxiety-Like Behaviors With the Alteration of Gut Microbiota
Source: Front Cell Infect Microbiol. 2021 Sep 14;11:697640. doi: 10.3389/fcimb.2021.697640 (PMC8476956; doi:10.3389/fcimb.2021.697640)
Supplement: Supplementary file 5 [file Table_3.docx]

**Table S3.** The 27 discriminative ASVs among CON+Saline, CUMS+Saline and CUMS+Inosine groups.

| **Species name** | **Family** | **Phylum** | **CUMS**  **stress** | **Inosine treatment** |
| --- | --- | --- | --- | --- |
| **ASV585** | Muribaculaceae | Bacteroidota | Down-regulated (LAD=2.84, P=0.01) | Up-regulated (LAD=2.61, P=0.03) |
| **ASV36** | Staphylococcaceae | Firmicutes | Down-regulated (LAD=3.51, P＜0.01) | Up-regulated (LAD=3.09, P=0.01) |
| **ASV578** | Muribaculaceae | Bacteroidota | Down-regulated (LAD=3.07, P=0.03) | Up-regulated (LAD=2.60, P=0.03) |
| **ASV564** | Muribaculaceae | Bacteroidota | Down-regulated (LAD=3.12, P=0.02) | Up-regulated (LAD=3.07, P=0.01) |
| **ASV561** | Muribaculaceae | Bacteroidota | Down-regulated (LAD=3.22, P=0.02) | Up-regulated (LAD=2.78, P<0.01) |
| **ASV14** | Erysipelotrichaceae | Firmicutes | Down-regulated (LAD=3.19, P=0.02) | Up-regulated (LAD=2.98, P=0.03 |
| **ASV510** | Rikenellaceae | Bacteroidota | Down-regulated (LAD=2.68, P=0.04) | Up-regulated (LAD=2.72, P=0.04) |
| **ASV70** | Muribaculaceae | Bacteroidota | Down-regulated (LAD=4.09, P=0.01) | Up-regulated (LAD=3.38, P=0.02) |
| **ASV71** | Lachnospiraceae | Firmicutes | Down-regulated (LAD=2.75, P=0.03) | Up-regulated (LAD=3.03, P<0.01) |
| **ASV431** | Muribaculaceae | Bacteroidota | Down-regulated (LAD=2.84, P=0.01) | Up-regulated (LAD=2.90, P=0.01) |
| **ASV232** | Oscillospiraceae | Firmicutes | Down-regulated (LAD=2.69, P=0.03) | Up-regulated (LAD=2.93, P=0.04) |
| **ASV63** | Bacteroidaceae | Bacteroidota | Down-regulated (LAD=3.23, P=0.03) | Up-regulated (LAD=3.35, P=0.03) |
| **ASV65** | Sutterellaceae | Proteobacteria | Down-regulated (LAD=3.20, P<0.01) | Up-regulated (LAD=2.78, P=0.05) |
| **ASV41** | Muribaculaceae | Bacteroidota | Down-regulated (LAD=3.05, P=0.04) | Up-regulated (LAD=3.13, P<0.01) |
| **ASV910** | Bacteroidaceae | Bacteroidota | Down-regulated (LAD=2.65, P=0.03) | Up-regulated (LAD=2.59, P=0.01) |
| **ASV97** | Rikenellaceae | Bacteroidota | Down-regulated (LAD=2.85, P<0.01) | Up-regulated (LAD=3.04, P=0.03) |
| **ASV591** | Muribaculaceae | Bacteroidota | Down-regulated (LAD=2.59, P= 0.01) | Up-regulated (LAD=2.75, P=0.01) |
| **ASV820** | Oscillospiraceae | Firmicutes | Up-regulated (LAD=2.75, P=0.03) | Down-regulated (LAD=2.94, P=0.03) |
| **ASV1** | Lactobacillaceae | Firmicutes | Up-regulated (LAD=4.83, P= 0.01) | Down-regulated (LAD=4.71, P=0.05) |
| **ASV803** | Norank_o_Clostridia_UCG-014 | Firmicutes | Up-regulated (LAD=2.75, P=0.03) | Down-regulated (LAD=2.68, P=0.03) |
| **ASV796** | Norank_o_Clostridia_vadinBB60_group | Firmicutes | Up-regulated (LAD=2.81, P=0.01) | Down-regulated (LAD=2.87, P=0.01) |
| **ASV60** | Saccharimonadaceae | Patescibacteria | Up-regulated (LAD=3.26, P=0.01) | Down-regulated (LAD=3.17, P=0.02) |
| **ASV404** | Bifidobacteriaceae | Actinobacteriota | Up-regulated (LAD=2.80, P=0.03) | Down-regulated (LAD=2.66, P=0.03) |
| **ASV407** | Monoglobaceae | Firmicutes | Up-regulated (LAD=2.93, P<0.01) | Down-regulated (LAD=2.78, P=0.02) |
| **ASV264** | Marinifilaceae | Bacteroidota | Up-regulated (LAD=3.78, P<0.01) | Down-regulated (LAD=3.71, P=0.01) |
| **ASV290** | Rikenellaceae | Bacteroidota | Up-regulated (LAD=3.85, P=0.01) | Down-regulated (LAD=3.92, P=0.05) |
| **ASV347** | Rikenellaceae | Bacteroidota | Up-regulated (LAD=2.91, P=0.01) | Down-regulated (LAD=2.79, P=0.04) |

Red: Up-regulated; Green: Down-regulated.
